# Clinical differences between respiratory viral and bacterial mono- and dual pathogen detected among Singapore military servicemen with febrile respiratory illness

**DOI:** 10.1111/irv.12312

**Published:** 2015-06-09

**Authors:** Zheng Jie Marc Ho, Xiahong Zhao, Alex R Cook, Jin Phang Loh, Sock Hoon Ng, Boon Huan Tan, Vernon J Lee

**Affiliations:** a HQ Medical Corps, Singapore Armed ForcesSingapore; b Saw Swee Hock School of Public Health, National University of SingaporeSingapore; c DSO National LaboratoriesSingapore

**Keywords:** epidemiology, military personnel, respiratory infections, surveillance

## Abstract

**Background:**

Although it is known that febrile respiratory illnesses (FRI) may be caused by multiple respiratory pathogens, there are no population-level studies describing its impact on clinical disease.

**Methods:**

Between May 2009 and October 2012, 7733 FRI patients and controls in the Singapore military had clinical data and nasal wash samples collected prospectively and sent for PCR testing. Patients with one pathogen detected (mono-pathogen) were compared with those with two pathogens (dual pathogen) for differences in basic demographics and clinical presentation.

**Results:**

In total, 45.8% had one pathogen detected, 20.2% had two pathogens detected, 30.9% had no pathogens detected, and 3.1% had more than two pathogens. Multiple pathogens were associated with recruits, those with asthma and non-smokers. Influenza A (80.0%), influenza B (73.0%) and mycoplasma (70.6%) were most commonly associated with mono-infections, while adenovirus was most commonly associated with dual infections (62.9%). Influenza A paired with *S. pneumoniae* had higher proportions of chills and rigors than their respective mono-pathogens (*P* = 0.03, *P* = 0.009). *H. influenzae* paired with either enterovirus or parainfluenzae had higher proportions of cough with phlegm than their respective mono-pathogens. Although there were observed differences in mean proportions of body temperature, nasal symptoms, sore throat, body aches and joint pains between viral and bacterial mono-pathogens, there were few differences between distinct dual-pathogen pairs and their respective mono-pathogen counterparts.

**Conclusion:**

A substantial number of FRI patients have multiple pathogens detected. Observed clinical differences between patients of dual pathogen and mono-pathogen indicate the likely presence of complex microbial interactions between the various pathogens.

## Introduction

Febrile respiratory illnesses (FRI) are caused by a wide range of pathogens, most commonly by viruses and bacteria,^1^,^2^ some of which cause more serious clinical disease and morbidity.^3^,^4^ It may also be due to multiple pathogens co-existing in a microenvironment of complex interactions,^5^ which is not unexpected as the respiratory mucosa has abundant resident flora to begin with. For instance, one study showed that 15.3% of ambulatory patients with influenza-like illness had two viruses detected,^6^ and another found that in 28.2% of children with community-acquired pneumonia, the illness was due to mixed viral–bacterial infections.^7^ Others also previously described respiratory viral^8^,^9^ and bacterial co-infections^10^,^11^ in various settings, although most focus on specific pathogen combinations, especially of the synergism between influenza and Streptococcus pneumoniae (*S. pneumoniae*).^12^–^15^

However, there are no population-level studies describing multiple pathogens among persons with upper respiratory tract infections and their impact on clinical disease. Such information is of particular importance to countries within the tropical belt where there is a predilection towards multiple pathogens due to the year-round circulation of respiratory pathogens.^16^,^17^

A previous study documented the clinical characteristics and epidemiology of viral mono-pathogens gleaned from the respiratory disease sentinel surveillance programme of the Singapore military.^18^ Here, we analyse additional data from the programme, compare patients with one (mono-pathogen) and two pathogens detected (dual pathogen), and describe observed differences in clinical characteristics.

## Methods

### Study site and population

All Singaporean males enter national service for 2 years after high school or equivalent. During this period, the majority spend most of their time in communal living and training quarters in military camps and return home on weekends, resulting in a semi-closed environment with community interaction.

Sentinel surveillance for febrile respiratory patients were performed at five major sites. The period of study was from May 2009 to Oct 2012, and servicemen who sought primary health care at these camps during regular consultation hours were recruited. The FRI inclusion criterion was having a body temperature of 37.5^°^C and above with cough or sore throat. After obtaining informed consent, a standardised questionnaire was administered and nasal wash sampling performed by trained personnel followed by routine clinical assessment by an attending physician. Repeat consultations were excluded if the patient was deemed to not have recovered from the first episode of illness.

Two weeks after the initial consultation, patients were reviewed (through case records and phone calls to patients, if necessary) to determine the number of patients who eventually required referral to hospitals for further evaluation, were diagnosed with pneumonia and/or were admitted for further treatment.

Randomly selected unmatched controls (at a rate of 5–10 persons per week) were also obtained across the year for comparative purposes of baseline commensal rates: these are soldiers from the same camps who were reporting sick at the medical centre for reasons other than respiratory symptoms or acute infections (e.g. those with muscle sprains were selected as controls). This is to prevent mild respiratory infections from being selected and confounding the baseline rates. Informed consent was also sought from controls before recruitment.

### Laboratory methods

Nasal wash samples were obtained from trained medical staff from each side of the nose and placed in universal transport media. These were stored in fridge at 4^°^C and transported to the laboratory using carriers with ice packs within 24 h.

An ISO15189-accredited laboratory that regularly takes part in QCMD EQA programmes was used to perform molecular diagnostic testing. Detailed laboratory methods have been described in the previous publication.^18^ Briefly, this was done by the extraction of nucleic acids using the DNA mini kit (Qiagen, Inc, Valencia, CA, USA) and then tested using multiplex PCR assays coupled with bead array detection technology (Resplex I and II, version 2.0, Qiagen, Inc, Valencia, CA, USA) which can simultaneously detect and subtype 18 different pathogens.

### Statistical methods

First, pathogens of the same genus were grouped (e.g. ‘influenza A’ includes its various subtypes, and ‘enterovirus’ also includes coxsackievirus, echovirus and rhinovirus). Demographic characteristics for controls, mono-pathogens, dual pathogens and patients with more than two pathogens were analysed and compared using descriptive statistics. Analyses on the prevalence of co-existing pathogens were then performed. Interval/ratio variables were compared using one-way analysis of variance (one-way ANOVA). Comparison of nominal variables with expected frequencies less than or equal to 5 was done using Fisher's exact test, while comparison of nominal variables with expected frequencies more than 5 was done using Pearson's chi-square test. Pearson's chi-square test was conducted to identify trend in proportions.

Further analysis focussed on comparing patients with one and two pathogens. In this regard, i) controls, ii) patients with more than two pathogens as well as iii) mono- and dual pathogens with sample sizes of less than 15 observations (considered too small for analysis) were excluded. As a result, a total of 11 mono-pathogens and 18 dual-pathogen pairs were available for comparison.

Permutation tests were conducted to compare the number of symptoms observed between mono-pathogen and dual-pathogen patients for each pathogen as a proxy for severity of infection. To assess differences in symptom expression, dual pathogens were compared against mono-pathogens for mean proportions of 16 symptoms (or signs). Empirical proportions of symptoms with 95% confidence intervals (CIs) for both mono-pathogens and dual pathogens were calculated and compared using Pearson's chi-square test at a significance level of 0.05. Symptoms with onsets in at least 30% of patients for a minimum of one pathogen or combination were described in detail. In particular, dual infections with statistically different results from their respective viral mono-infections were highlighted.

R Statistical Software (version 3.0.3) was used to perform all statistical analyses.^19^

Ethics approval was given by the Singapore military Joint Medical Committee for Research and the National University of Singapore's ethics review committee.

## Results

### Number of pathogens detected

Of 7733 samples of patients tested, 45.8% had mono-pathogens and 20.2% had dual pathogens detected. No pathogens were picked up in 30.9% samples, while 3.1% samples had more than two pathogens. Among dual pathogens, virus–bacterial pairs were most common at 76.0%, followed by bacteria–bacteria (15.2%) and virus–virus pairs (8.8%).

### Demographics

Demographics for patients and controls are detailed in Table1. Gender and the prevalence of heart disease were similar across all groups. Mean age was slightly higher in controls, and the number of persons with asthma was higher among patients. Multiple pathogens were also more commonly detected among recruits and in those not currently smoking.

**Table 1:** Demographics of FRI patients and controls. By Kruskal–Wallis test, comparing median age across all groups; by Fisher's exact test, comparing proportions of gender and having heart disease across all groups; and by Person's chi-square test, comparing proportions of all the other characteristics across all groups. *P*-values compared across all columns, with any statistical significance reflected

Characteristic	Controls	Patients				p-value
		No pathogens	Mono-pathogens	Dual pathogens	>2 pathogens	
Median age (Range)	20.6 (17.6, 55.0)	20.4 (17.3, 59.4)	20.3 (13.2, 60.0)	20.2 (17.7, 50.7)	20.3 (17.4, 37.1)	<0.001
Male (%)	1342 (99.9)	2382 (99.6)	3532 (99.8)	1563 (99.9)	236 (99.2)	0.10
Recruit (%)	480 (35.7)	1447 (60.5)	2562 (72.4)	1344 (84.9)	215 (90.3)	<0.001
Current smoker (%)	385 (28.6)	760 (31.8)	923 (26.1)	358 (22.9)	49 (20.6)	<0.001
Asthma (%)	236 (17.6)	447 (18.7)	748 (21.1)	352 (22.5)	50 (21.0)	0.003
Heart disease (%)	19 (1.4)	30 (1.3)	40 (1.1)	11 (0.7)	2 (0.8)	0.39
Total (%)	1344	2391	3540	1564	238	–
	7733					

### Breakdown of pathogens detected

**Figure 1:**
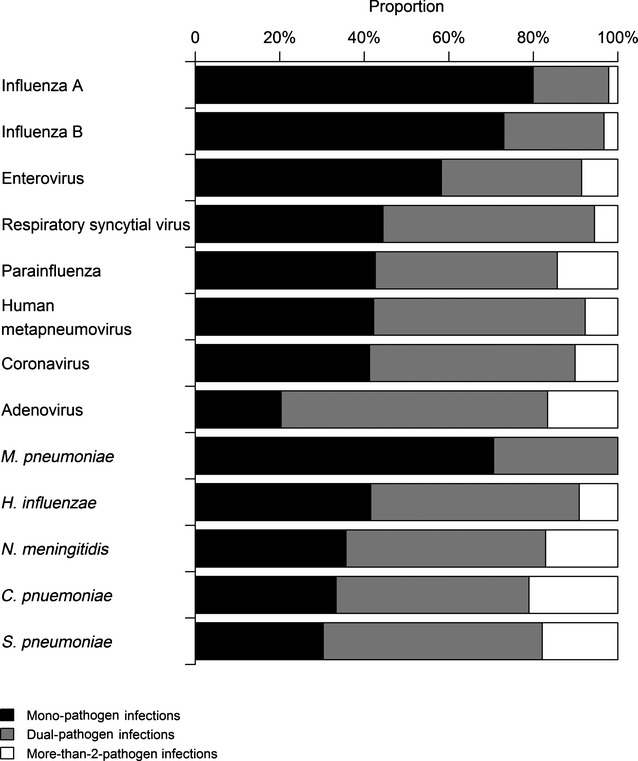
Proportion of mono-, dual- and more than 2 pathogens per pathogen. The pathogens are listed vertically, viruses followed by bacteria. The horizontal axis measures the proportion of mono-pathogens, dual pathogens and more than 2 pathogens per pathogen. Mono-infections are in black, dual pathogens are in grey, and more than 2 pathogens are in white. The length of the polygon represents the magnitude of each proportion.

Figure1 details proportions of mono-, dual and more than 2 pathogens detected for each pathogen. Influenza A (80.0%), influenza B (73.0%) and *Mycoplasma pneumoniae* (*M. pneumoniae*) (70.6%) tended to occur alone. Adenovirus was most likely to occur as part of a dual-pathogen infection (63.0%).

Table2 shows the differences in detection of pathogens between patients and controls. There were no significant differences in RSV, *M. pneumonia*, *S. pneumonia* and *N. meningitidis* between the two groups.

**Table 2:** Detection of pathogens between patients and controls. By Fisher's exact test, comparing detection of each pathogen among patients and controls

Pathogen	Patients (*n* = 7733)		Controls (*n* = 1344)		*P*-value
	*n*	%	*n*	%	
Influenza A	773	10.0	16	1.2	<0.001
Influenza B	604	7.8	7	0.5	<0.001
RSV	18	0.2	1	0.1	0.34
Parainfluenzae	209	2.7	2	0.1	<0.001
hMPV	142	1.8	0	0	<0.001
Enterovirus	1236	16.0	61	4.5	<0.001
Adenovirus	644	8.3	13	1	<0.001
Coronavirus	405	5.2	17	1.3	<0.001
*M. pneumoniae*	17	0.2	2	0.1	1
*C. pneumoniae*	138	1.8	8	0.6	<0.001
*S. pneumoniae*	637	8.2	128	9.5	0.12
*N. meningitidis*	199	2.6	46	3.4	0.08
*H. influenzae*	2367	30.6	188	14.0	<0.001

**Figure 2:**
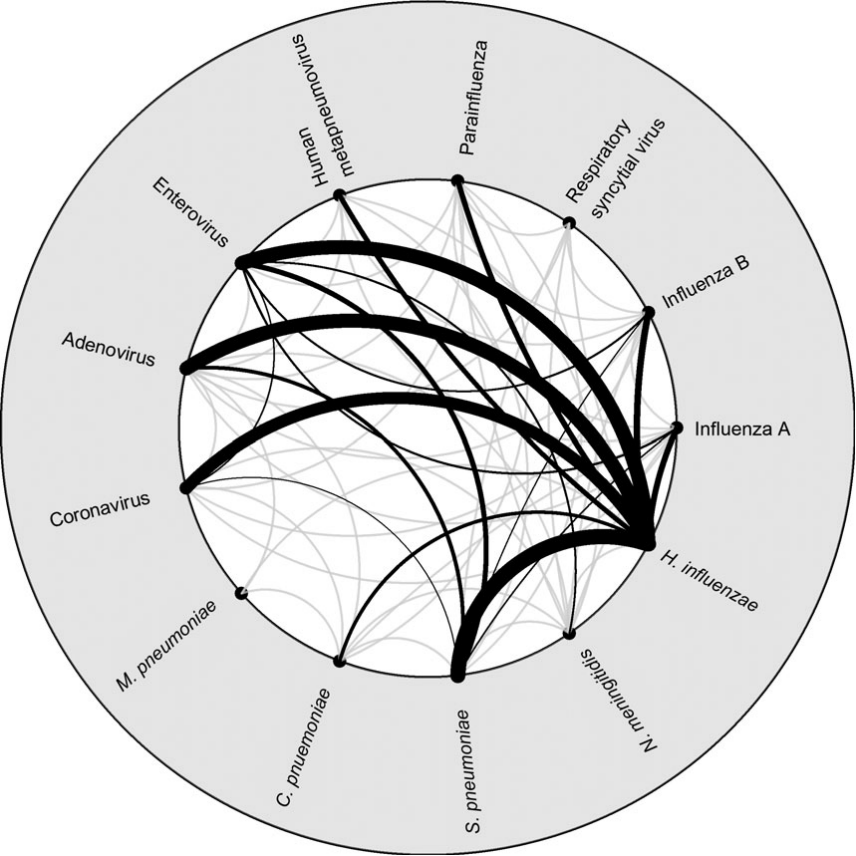
Number of observations per pair of pathogens (dual pathogens). Each point represents a pathogen. A curve is drawn between two pathogens if there is a patient with both pathogens detected. Curves in black represent dual pathogens with counts of more than or equal to 15, while curves in grey represent dual pathogens with counts of less than 15 patients. The thickness of each curve represents the number of patients (thin indicates less; thick indicates more).

Among dual pathogens, there were 13 virus–bacteria, 2 bacteria–bacteria and 3 virus–virus combinations with more than 15 observations each. The most common virus–virus pair was that of influenza A with enterovirus; and of bacteria–bacteria pairs, it was *Haemophilus influenzae* (*H. influenzae*) with *S. pneumoniae*. The top three virus–bacteria observations were *H. influenzae*, paired with adenovirus, enterovirus and coronavirus, respectively. Figure2 depicts the incidence of dual-pathogen pairs, with further details in Table S1.

Of the 238 samples with more than 2 pathogens detected, *H. influenzae*, *S. pneumoniae*, adenovirus and enterovirus were most commonly involved. The most common trio was adenovirus with *S. pneumoniae* and *H. influenzae*, which accounted for 15.1% of samples with more than 2 pathogens.

### Correlations with symptoms

#### Number of symptoms

**Figure 3:**
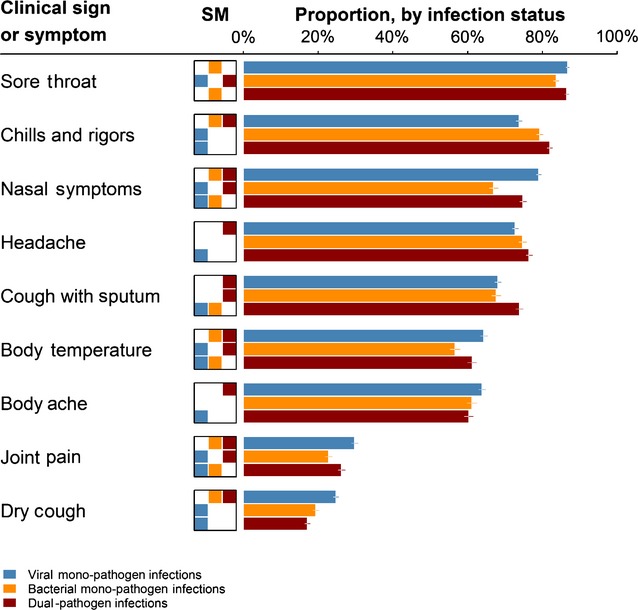
Univariate comparison of clinical signs or symptoms among viral mono-pathogen, bacterial mono-pathogen and dual pathogens. The bars with whiskers, which represent 95% confidence intervals, in the right column indicate empirical frequencies of clinical signs or symptoms for different viruses. The significance matrix (SM) in the centre indicates the significant differences in symptoms for pairs of types of infections. For example, for a headache, viral mono-pathogen is significantly different from dual pathogens indicated by the dark red cell in the row for viral mono-pathogen and the steel blue cell in the row for dual pathogens.

Mono- and dual-pathogen patients had similar symptom loads (with 8.3 symptoms on average). However, among dual-pathogen patients, those involving *S. pneumoniae* (*P* = 0.02), Neisseria meningitidis (*N. meningitidis*) (*P* = 0.01) and *H. influenzae* (*P* = 0.02) displayed a higher number of symptoms than corresponding mono-pathogen patients. Nine common symptoms, not ranked by severity, are presented in Figure3, with further details in Table S2 and Figure S1.

#### Body temperature

Mean body temperature of viral mono-pathogen patients was slightly higher than that of bacterial mono-pathogen patients (38.2^°^C vs 38.1^°^C, *P* < 0.001). Dual-pathogen patients involving *S. pneumoniae* with either influenza A (38.5^°^C, 95%CI 38.2, 38.7) or influenza B (38.7^°^C, 95%CI 38.3, 39.0) had the highest mean body temperatures, although these were not significantly different from respective mono-pathogens.

#### Chills and rigours

Mean proportion of viral mono-pathogen patients with chills and rigors was lower than that of bacterial mono-pathogen patients (0.737 vs 0.793; *P* < 0.001). Dual pathogens with both *S. pneumoniae* and influenza A were associated with high proportions of chills and rigors (1.00, 95%CI 0.805, 1.00). This was significantly more than *S. pneumoniae* (0.767, 95%CI 0.701, 0.825; *P* = 0.03) and influenza A (0.728, 95%CI 0.691, 0.763; *P* = 0.009) mono-pathogen patients.

#### Cough with sputum

Mean proportions of viral and bacterial mono-pathogen patients having cough with sputum were similar, at 0.681 and 0.677, respectively, although mean proportion of dual pathogens with the symptom was higher, at 0.738 (*P* < 0.001). Specific dual pathogens with a higher proportion of cough with sputum than both respective bacterial and viral mono-pathogen patients were *H. influenzae* paired with enterovirus (*P* = 0.002; *P* = 0.001), or parainfluenzae (*P* = 0.02; *P* = 0.002).

#### Dry cough

Mean proportion of viral mono-pathogen patients having dry cough was higher than that of bacterial mono-pathogen patients (0.249 vs 0.194; *P* < 0.001). *H. influenzae* with enterovirus, with higher mean proportions of cough with phlegm as described above, showed a corresponding decrease in dry cough. The proportion among dual-pathogen patients was also lower than the patients infected with the virus alone or the bacteria alone (*P* = 0.005, *P* = 0.03, respectively).

#### Nasal symptoms

Mean proportion of viral mono-pathogen patients having nasal symptoms (sneezing, blocked nose and running nose) was higher than that of bacterial mono-pathogen patients (0.790 vs 0.670, *P* < 0.001). Mean proportion for dual infections with nasal symptoms lay in between at 0.748, statistically different from both viral (*P* = 0.002) and bacterial (*P* < 0.001) mono-pathogen levels. However, no specific dual-pathogen pairs had statistically different levels than their respective viral mono-pathogens.

#### Sore throat

Mean proportion of viral mono-pathogen patients with sore throat was only slightly higher than that of bacterial mono-pathogen patients (0.868 vs 0.837; *P* = 0.01). The mean proportions for dual pathogens were similar to viral mono-pathogen levels (0.865) and likewise statistically higher than bacterial mono-pathogen levels (*P* = 0.03). Interestingly, however, dual pathogens of coronavirus with *S. pneumoniae* (*P* = 0.007) or *H. influenzae* (*P* = 0.001) were instead found to be statistically lower than patients with coronavirus alone.

#### Headache

Mean proportions of viral mono-pathogen patients with headache were similar to that of bacterial mono-pathogen patients (0.727 vs 0.747). For dual pathogens (0.765), the mean proportion were only slightly higher than viral mono-pathogen patients (*P* = 0.01). However, no dual-pathogen pairs had statistically different levels than their respective viral mono-pathogens.

#### Body aches

Mean proportions of viral mono-pathogen patients with body aches were similar to that of bacterial mono-pathogen patients (0.638 vs 0.611). Mean proportion of dual pathogens with body aches (0.603) was slightly lower than viral mono-pathogen patients (*P* = 0.03). However, no dual-pathogen pairs had statistically different levels than their respective viral mono-pathogens.

#### Joint pains

Mean proportion of viral mono-pathogen patients with joint pains were higher than that of bacterial mono-pathogen patients (0.298 vs 0.228; *P* ≤ 0.001). Mean proportions of dual infections with joint pains (0.263) were in between these two levels, being statistically different from both viral (*P* = 0.02) and bacterial (*P* = 0.03) mono-pathogen patients. However, no dual-pathogen pairs had statistically different levels than their respective viral mono-pathogens.

#### Pneumonia

Patients were reviewed 2 weeks after the first consultation to ascertain whether any complications had developed in the interim. The proportion of patients referred to hospitals (for further evaluation), as well as the proportion diagnosed with pneumonia, were found to increase significantly with the number of pathogens detected (*P* = 0.001 and *P* = 0.04, respectively) (Table3). However, there were no clear trends in the number of patients eventually requiring inpatient treatment, possibly as a result of relatively small numbers.

**Table 3:** Referrals to hospitals, diagnoses of pneumonia and location of treatment of FRI patients within 2 weeks of initial consultation. By Fisher's exact test, comparing across groups

	Referred to Hospital for evaluation		Diagnosed as pneumonia		Outpatient Treatment for pneumonia		Inpatient Treatment for pneumonia	
	*n*	%	*n*	%	*n*	%	*n*	%
No pathogen (*n* = 2391)	141	5.90	8	0.33	5	0.21	3	0.12
Mono-pathogen (*n* = 3540)	226	6.38	20	0.56	5	0.14	15	0.42
Viral (*n* = 1305)	119	9.12	6	0.46	2	0.15	4	0.31
Bacterial (*n* = 2235)	107	4.79	14	0.63	3	0.13	11	0.49
Dual pathogen (*n* = 1564)	122	7.80	13	0.83	9	0.58	4	0.26
Viral–Viral (*n* = 138)	9	6.52	0	0	0	0	0	0
Viral–Bacterial (*n* = 1189)	93	7.82	9	0.76	5	0.42	4	0.34
Bacterial–Bacterial (*n* = 237)	20	8.44	4	1.69	4	1.69	0	0
More than 2 pathogens (*n* = 238)	28	11.76	2	0.84	1	0.42	1	0.42
Total (*n* = 7733)	517	6.69	43	0.55	20	0.26	23	0.30
*P*-value	0.002		0.14		0.04		0.14	

## Discussion

Much emphasis in respiratory illness research that is based on clinical presentations has thus far centred on mono-infections, although in reality a substantial portion of patients may actually have two or more potential pathogens. Our study shows that the prevalence of patients with two or more pathogens in a tropical setting was 23.3%, most commonly due to virus–bacteria pairs. Often, it seems that the role of ‘less pathogenic’ co-detected microbes are casually disregarded – perhaps for ease of data interpretation. Yet such assumptions are questionable especially because the impact of multiple pathogens on clinical characteristics has not been well studied. This formed the impetus for our analysis of the distribution of dual pathogens in ambulatory FRI patients, and comparing associated clinical presentations between mono- and dual-pathogen patients.

Although we cannot conclude cause–effect relationships from the study, we noted a few interesting trends. The association between new recruits and multiple pathogens is likely due to the ease of transmission within the communal environment (of increased population density) on entry into military service, as described in clinical studies among similar cohorts.^20^,^21^ These conditions also promote shifts in predominant circulating respiratory pathogens with time, as had been previously described,^18^ sometimes culminating in outbreaks of respiratory disease.^21^,^22^ To prevent the occurrence of such incidents, mitigating measures – such as appropriate education on hand and respiratory hygiene – have been implemented.

The higher prevalence of asthma in patients and the decreased number of pathogens among current smokers may also reflect the effects of the two on the upper respiratory tract.^23^,^24^ For example, previous studies describe the effect of cigarette smoke in causing reduced competitive commensal organisms in the respiratory tract.^25^,^26^

Among dual infections, virus–virus pairs constitute only 3.0% of the entire data set, within the lower end of range of viral co-infection studies in ambulatory settings (1.73–15.3%).^6^,^8^,^9^ This may be due to local interactions between immune and microbial mechanisms preventing the occurrence of co-existing viral respiratory pathogens. Such negative correlations have been previously described,^27^ including the replacement of one virus with another when the former is removed from the general population through vaccinations.^18^ The genus Enterovirus was most prevalent (56.6%) among viral–viral pairs, similar to two other viral co-infection studies reporting rhinovirus rates of 49.3% and 69.5%.^9^,^28^

Virus–bacterial pairs were most common, with a significant proportion involving adenovirus, particularly paired with *H. influenzae* (59.8%). Such a finding had also been previously observed among hospitalised children, where 45% of those with adenovirus were co-infected with various bacteria.^29^ Previous chinchilla models on experimental otitis media also point towards possible synergisms between adenovirus and *H. influenzae*,^30^ although further studies are needed to conclusively determine whether such interactions exist in the upper respiratory tract.

When it came to symptoms, the increased incidences of chills and rigors and elevated body temperatures in influenza A and influenza B, respectively, when paired with *S. pneumoniae* correspond to previous studies showing the disposition to superinfection caused by the influenza virus on respiratory epithelium, in both laboratory and hospital studies.^31^–^35^ Our results show that these apply to ambulatory patients as well. However, we also noted that these systemic-type symptoms appeared to be distinct from localised upper respiratory tract symptoms (such as running nose and cough), which were not found to be significantly different from patients with influenza alone.

Next, a higher prevalence of cough with phlegm was correlated with a number of dual-pathogen combinations, all of which involved the bacteria *H. influenzae*. Although there are microbiological studies on the bacteria's interactions with rhinovirus,^36^,^37^ there is insufficient information to conclusively explain the observations noted with parainfluenzae, warranting further studies.

Finally, diversity in the impact of dual pathogens on clinical manifestations, as seen through the results of other symptoms, is likely indicative of complex and diverse microbial interactions between respiratory pathogens in the upper respiratory tract. Bosch *et al*. have detailed a number of known microbiologic mechanisms, including various modalities of synergisms and competition between species.^6^ These include pathogens that are usually associated with asymptomatic colonisation in healthy individuals (e.g. *S. pneumoniae* and *H. influenzae*), which are potentially pathogenic with shifts in the respiratory tract microenvironment – for instance, the introduction of new microbes.^38^–^40^ Many of these are not yet fully understood, and it is hoped that such epidemiological data may spur greater interest in co-pathogen microbiology research.

### Limitations

Our study does not explore patients infected with more than 2 pathogens and co-pathogen pairs with <15 observations. Although it identifies observed correlations between pairs and symptoms, it does not determine sequence of pathogens in relation to onset of symptoms or prove causality, which require further microbiological or case–control epidemiological research. Severity of symptoms other than fever was not determined, actual diagnoses by doctors were not analysed, and further differences in the actual clinical impact could not be observed.

Although statistically significant differences have been described, the clinical significance of these findings have to be considered alongside as small differences may not be easily translatable to clinical practice and the large number of statistical comparisons increase the chances of type I (i.e. false-positive) errors. The study predominantly involved young adult males, limiting the generalizability to other populations. It is also conducted in a tropical setting with a fairly constant climate; thus, the effect of such changes on symptomology (e.g. in a temperate country) cannot be determined.

By grouping pathogens of the same genus together in analysis, it is also not possible to determine whether specific subtypes are the cause for the observations made. We are unable to detect the presence of dual-pathogen patients involving two or more viruses from the same genus, especially within enteroviruses. Although we compared differences in the detection of organisms between patients and controls, we are unable to conclude on whether certain organisms (such as *N. meningitidis* and adenovirus) are actually commensals, and PCR is not the optimal method for diagnosis of bacterial infections.

## Conclusion

We have described the aetiology of dual pathogens causing FRI in the tropical setting and compared differences with mono-pathogens with regard to observed clinical manifestations. The presence of higher incidences of certain symptoms with specific pathogen pairs is indicative of underlying complex microbial interactions and affirms existing microbiological co-pathogen studies. However, many of these processes are still not well explored in existing literature, opening many opportunities for further research into this area.

## Supporting Information

**Figure S1.** Proportions of each clinical symptom between dual pathogens and their mono-pathogen counterparts.

**Table S1.** Checkerboard of dual pathogens detected among cases. The 18 dual-pathogen pairs for further analysis of symptoms (i.e. 15 observations or more) are in bold. **Table S2.** Mean proportions and comparisons between viral and bacterial mono-pathogens and dual pathogens.

## References

[b1] Monto AS, Sullivan KM (1993). Acute respiratory illness in the community. Frequency of illness and the agents involved. Epidemiol Infect.

[b2] Feikin DR, Njenga MK, Bigogo G (2012). Etiology and Incidence of viral and bacterial acute respiratory illness among older children and adults in rural western Kenya, 2007-2010. PLoS ONE.

[b3] Hong C-Y, Lin RTP, Tan ESL (2004). Acute respiratory symptoms in adults in general practice. Fam Pract.

[b4] Seah SG-K, Lim EA-S, Kok-Yong S (2010). Viral agents responsible for febrile respiratory illnesses among military recruits training in tropical Singapore. J Clin Virol.

[b5] Bosch AATM, Biesbroek G, Trzcinski K, Sanders EAM, Bogaert D (2013). Viral and bacterial interactions in the upper respiratory tract. PLoS Pathog.

[b6] Peci A, Winter A-L, Gubbay JB (2013). Community-acquired respiratory viruses and co-infection among patients of Ontario sentinel practices, April 2009 to February 2010. Influenza Other Respir Viruses.

[b7] Cantais A, Mory O, Pillet S (2014). Epidemiology and microbiological investigations of community-acquired pneumonia in children admitted at the emergency department of a university hospital. J Clin Virol.

[b8] Cui S-J, Shi W-X, Huang F, Pang X-H, Deng Y (2013). Co-infection cases of human common respiratory viruses in Beijing, 2010-2012. Braz J Infect Dis.

[b9] Drews AL, Atmar RL, Glezen WP, Baxter BD, Piedra PA, Greenberg SB (1997). Dual respiratory virus infections. Clin Infect Dis.

[b10] Chiu C-Y, Chen C-J, Wong K-S, Tsai M-H, Chiu C-H, Huang Y-C (2013). Impact of bacterial and viral coinfection on mycoplasmal pneumonia in childhood community-acquired pneumonia. J Microbiol Immunol Infect.

[b11] Thorburn K, Riordan A (2012). Pulmonary bacterial coinfection in infants and children with viral respiratory infection. Expert Rev Anti Infect Ther.

[b12] Dawood FS, Chaves SS, Pérez A (2014). Complications and associated bacterial co-infections among children hospitalized with seasonal or pandemic influenza, United States, 2003-2010. J Infect Dis.

[b13] Cillóniz C, Ewig S, Menéndez R (2012). Bacterial co-infection with H1N1 infection in patients admitted with community acquired pneumonia. J Infect.

[b14] Blyth CC, Webb SAR, Kok J (2013). The impact of bacterial and viral co-infection in severe influenza. Influenza Other Respir Viruses.

[b15] Dhanoa A, Fang NC, Hassan SS, Kaniappan P, Rajasekaram G (2011). Epidemiology and clinical characteristics of hospitalized patients with pandemic influenza A (H1N1) 2009 infections: the effects of bacterial coinfection. Virol J.

[b16] Chew FT, Doraisingham S, Ling AE, Kumarasinghe G, Lee BW (1998). Seasonal trends of viral respiratory tract infections in the tropics. Epidemiol Infect.

[b17] Shek LP-C, Lee B-W (2003). Epidemiology and seasonality of respiratory tract virus infections in the tropics. Paediatr Respir Rev.

[b18] Tan XQ, Zhao X, Lee VJ (2014). Respiratory viral pathogens among Singapore military servicemen 2009-2012: epidemiology and clinical characteristics. BMC Infect Dis.

[b19] R core Team (2014). R: A Language and Environment for Statistical Computing.

[b20] White DW, Feigley CE, McKeown RE, Hout JJ, Hebert JR (2011). Association between barracks type and acute respiratory infection in a gender integrated Army basic combat training population. Mil Med.

[b21] Jeger V, Dünki A, Germann M (2011). H1N1 outbreak in a Swiss military boot camp–observations and suggestions. Swiss Med Wkly.

[b22] Cosby MT, Pimentel G, Nevin RL (2013). Outbreak of H3N2 influenza at a US military base in Djibouti during the H1N1 pandemic of 2009. PLoS ONE.

[b23] Tantilipikorn P, Auewarakul P (2011). Airway allergy and viral infection. Asian Pac J Allergy Immunol.

[b24] Kloepfer KM, Olenec JP, Lee WM (2012). Increased H1N1 Infection Rate in Children with Asthma. Am J Respir Crit Care Med.

[b25] Feldman C, Anderson R (2013). Cigarette smoking and mechanisms of susceptibility to infections of the respiratory tract and other organ systems. J Infect.

[b26] Brook I, Gober AE (2005). Recovery of potential pathogens and interfering bacteria in the nasopharynx of smokers and nonsmokers. Chest.

[b27] Wang Z, Malanoski AP, Lin B (2010). Broad spectrum respiratory pathogen analysis of throat swabs from military recruits reveals interference between rhinoviruses and adenoviruses. Microb Ecol.

[b28] Goka EA, Vallely PJ, Mutton KJ, Klapper PE (2014). Single, dual and multiple respiratory virus infections and risk of hospitalization and mortality. Epidemiol Infect.

[b29] Korppi M, Leinonen M, Mäkelä PH, Launiala K (1991). Mixed infection is common in children with respiratory adenovirus infection. Acta Paediatr Scand.

[b30] Suzuki K, Bakaletz LO (1994). Synergistic effect of adenovirus type 1 and nontypeable Haemophilus influenzae in a chinchilla model of experimental otitis media. Infect Immun.

[b31] Metzger DW, Sun K (2013). Immune dysfunction and bacterial coinfections following influenza. J Immunol.

[b32] McCullers JA (2006). Insights into the interaction between influenza virus and pneumococcus. Clin Microbiol Rev.

[b33] Peltola VT, Murti KG, McCullers JA (2005). Influenza virus neuraminidase contributes to secondary bacterial pneumonia. J Infect Dis.

[b34] Diavatopoulos DA, Short KR, Price JT (2010). Influenza A virus facilitates Streptococcus pneumoniae transmission and disease. FASEB J.

[b35] Short KR, Habets MN, Hermans PWM, Diavatopoulos DA (2012). Interactions between Streptococcus pneumoniae and influenza virus: a mutually beneficial relationship?. Future Microbiol.

[b36] Sajjan US, Jia Y, Newcomb DC (2006). *H. influenzae* potentiates airway epithelial cell responses to rhinovirus by increasing ICAM-1 and TLR3 expression. FASEB J.

[b37] Zaheer RS, Wiehler S, Hudy MH (2014). Human rhinovirus-induced ISG15 selectively modulates epithelial antiviral immunity. Mucosal Immunol.

[b38] García-Rodríguez JA, Fresnadillo Martínez MJ (2002). Dynamics of nasopharyngeal colonization by potential respiratory pathogens. J Antimicrob Chemother.

[b39] Brogden KA, Guthmiller JM, Taylor CE (2005). Human polymicrobial infections. Lancet.

[b40] Margolis E, Yates A, Levin BR (2010). The ecology of nasal colonization of Streptococcus pneumoniae, Haemophilus influenzae and *Staphylococcus aureus*: the role of competition and interactions with host's immune response. BMC Microbiol.

