# Supplementary figures and images for: Clinical differences between respiratory viral and bacterial mono- and dual pathogen detected among Singapore military servicemen with febrile respiratory illness
Source: Influenza Other Respir Viruses. 2015 Jun 9;9(4):200–8. doi: 10.1111/irv.12312 (PMC4474496; doi:10.1111/irv.12312)

## Slide 1
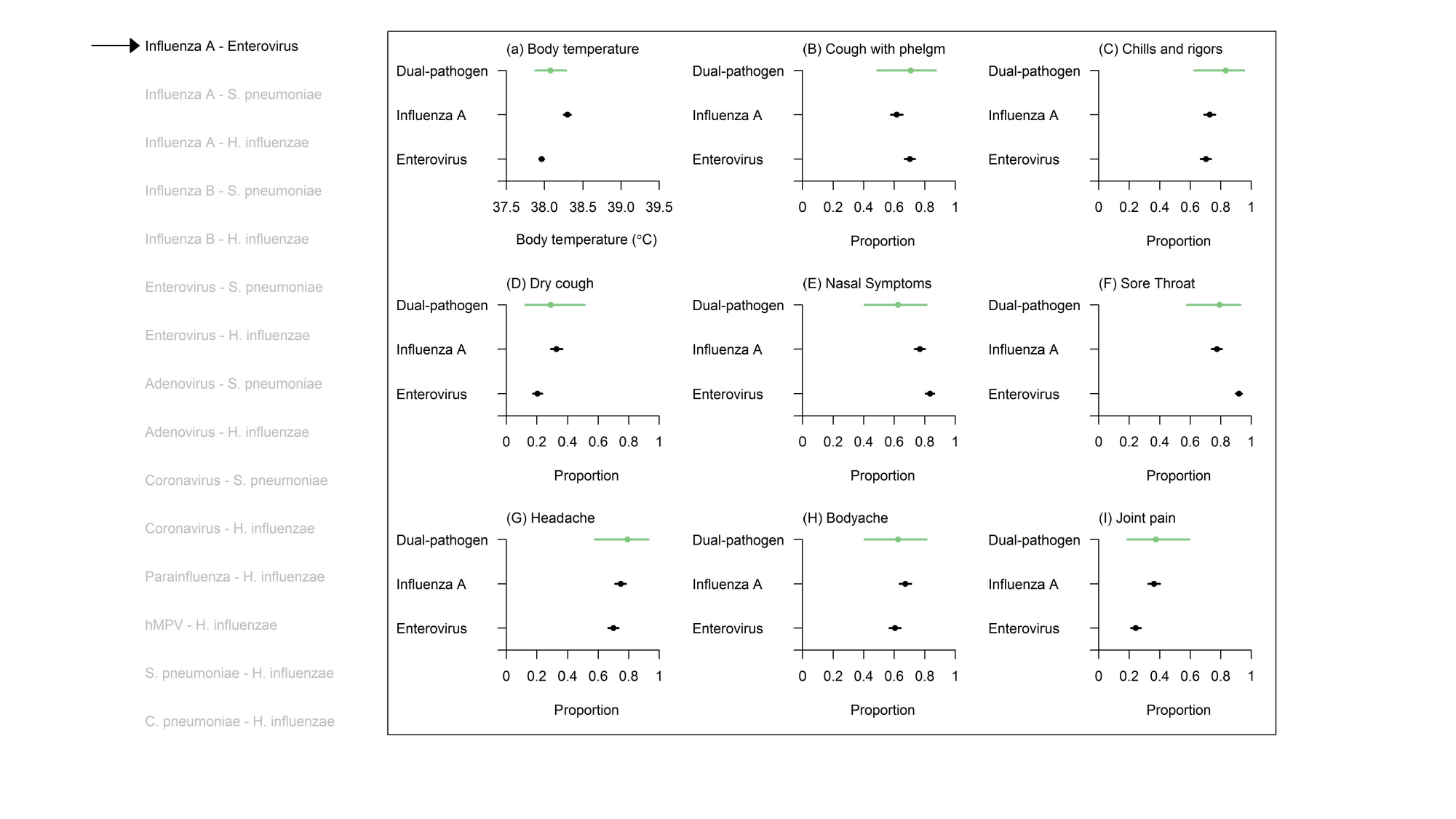

## Slide 2
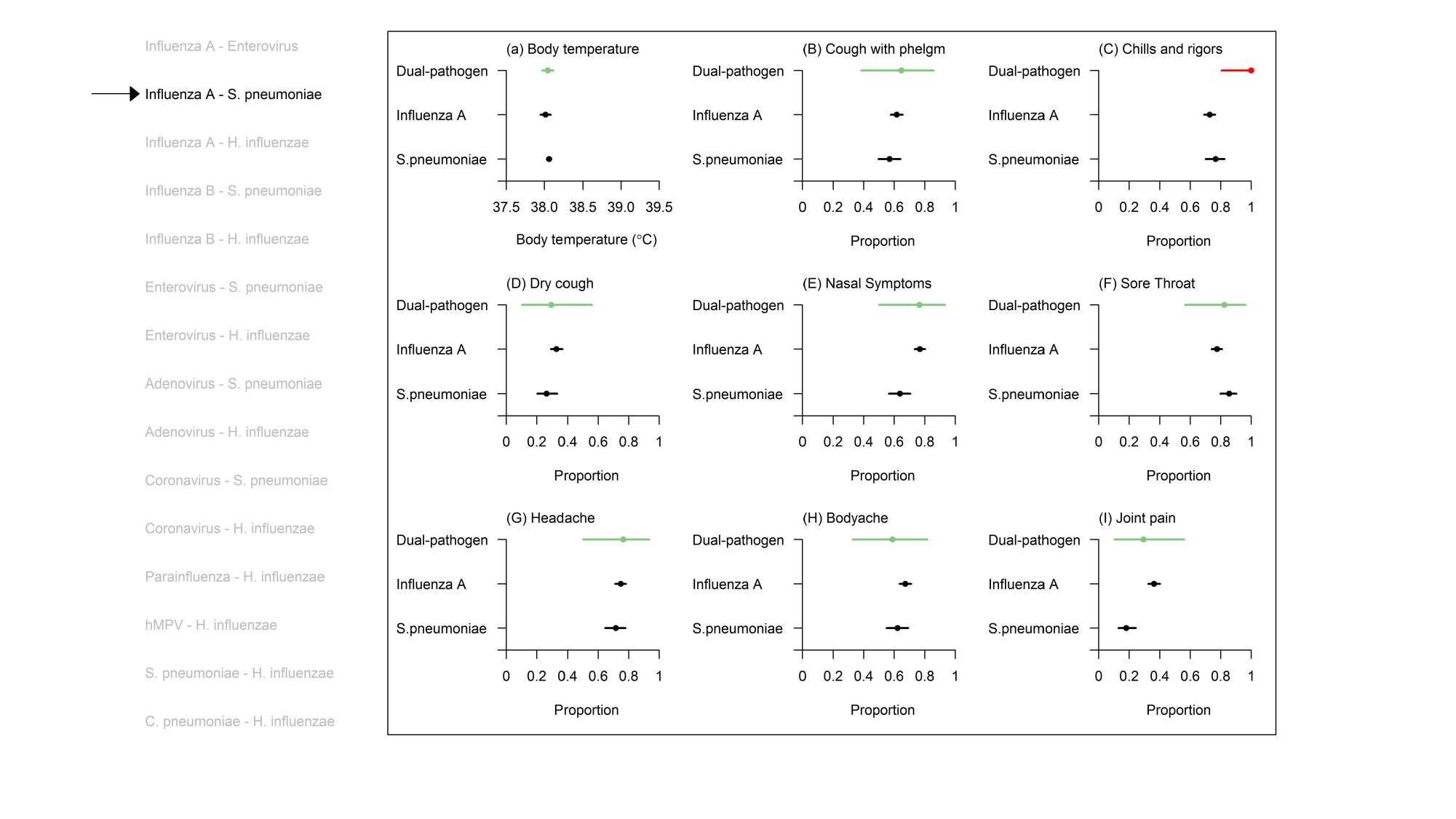

## Slide 3
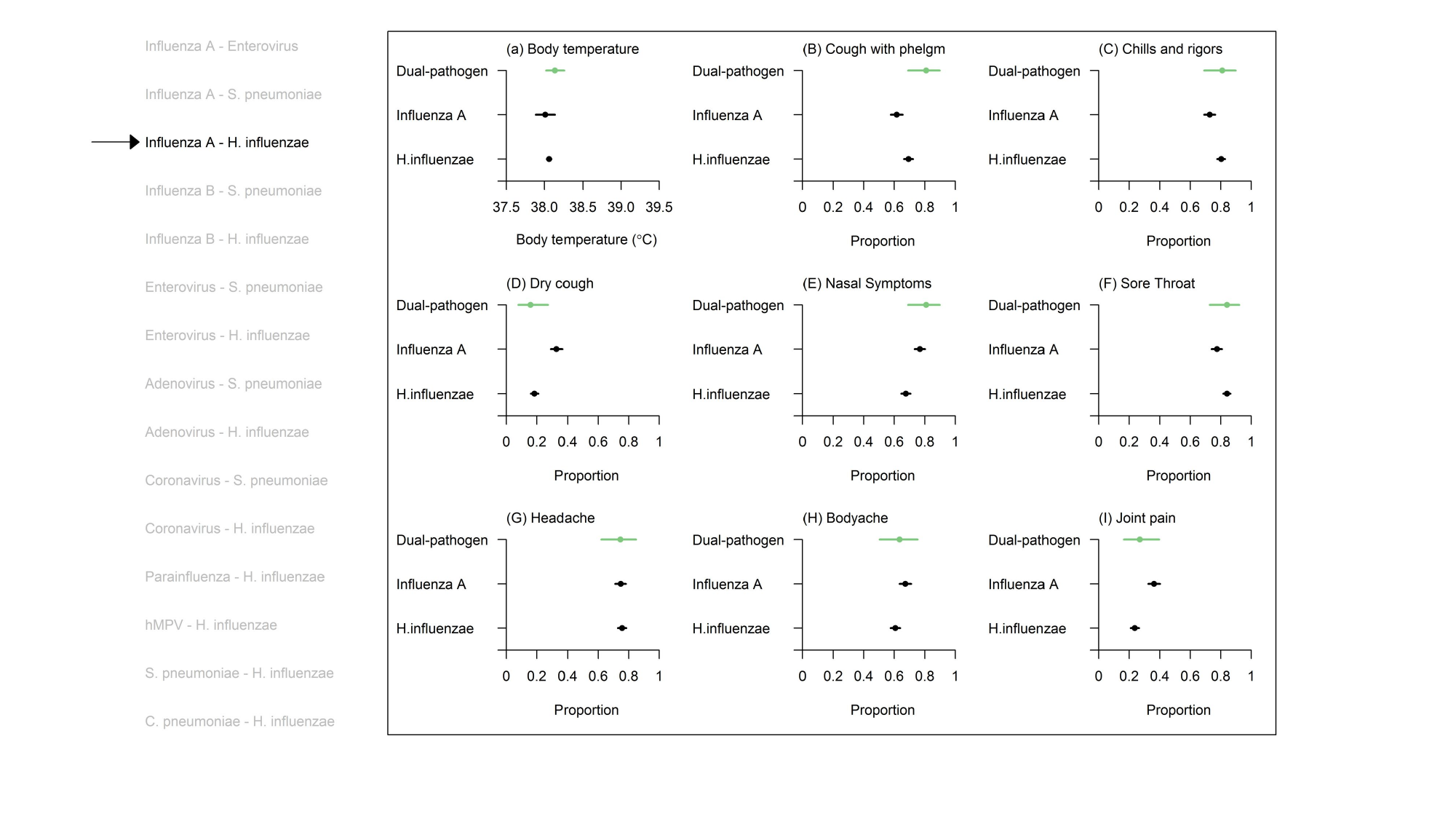

## Slide 4
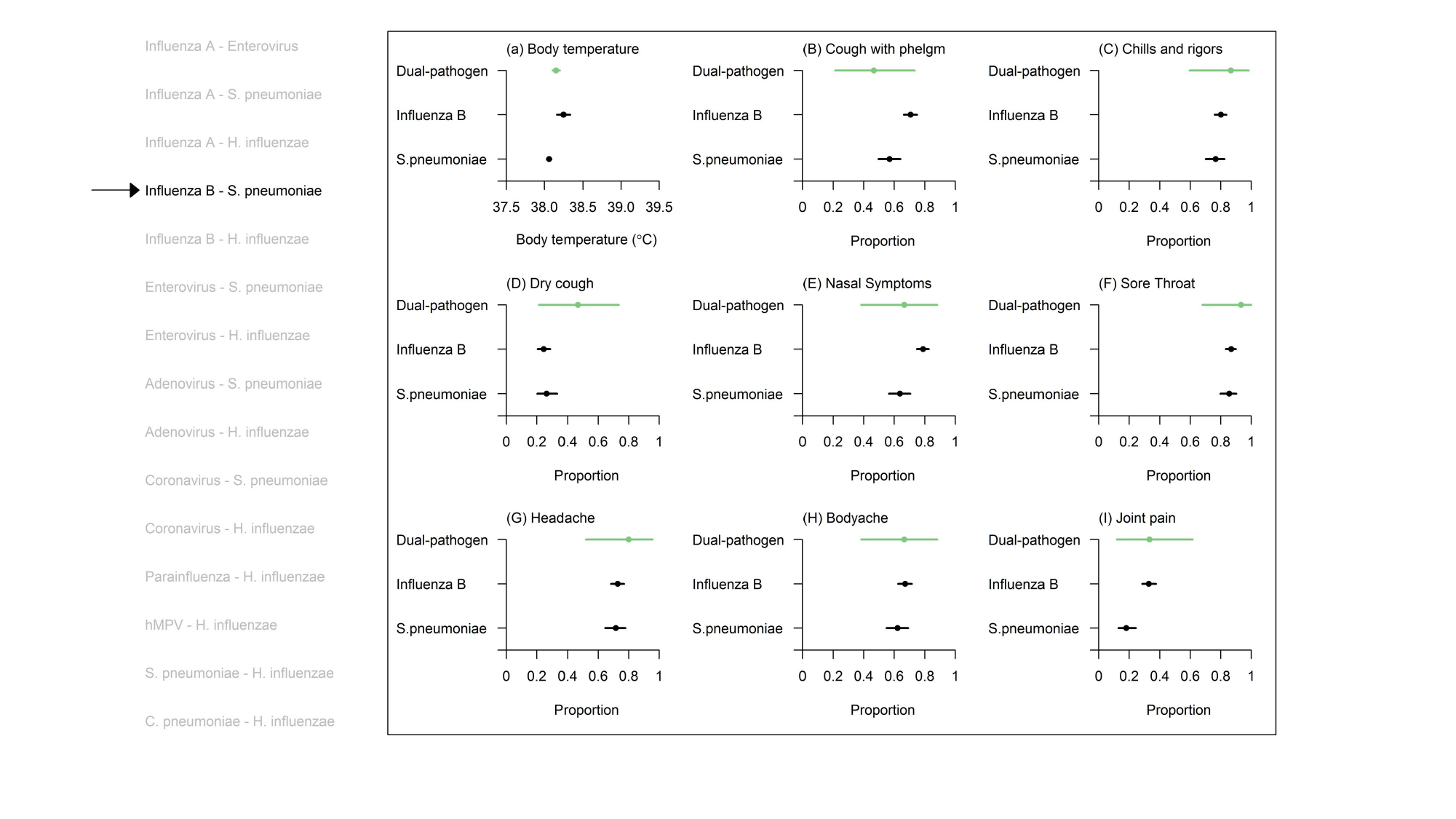

## Slide 5
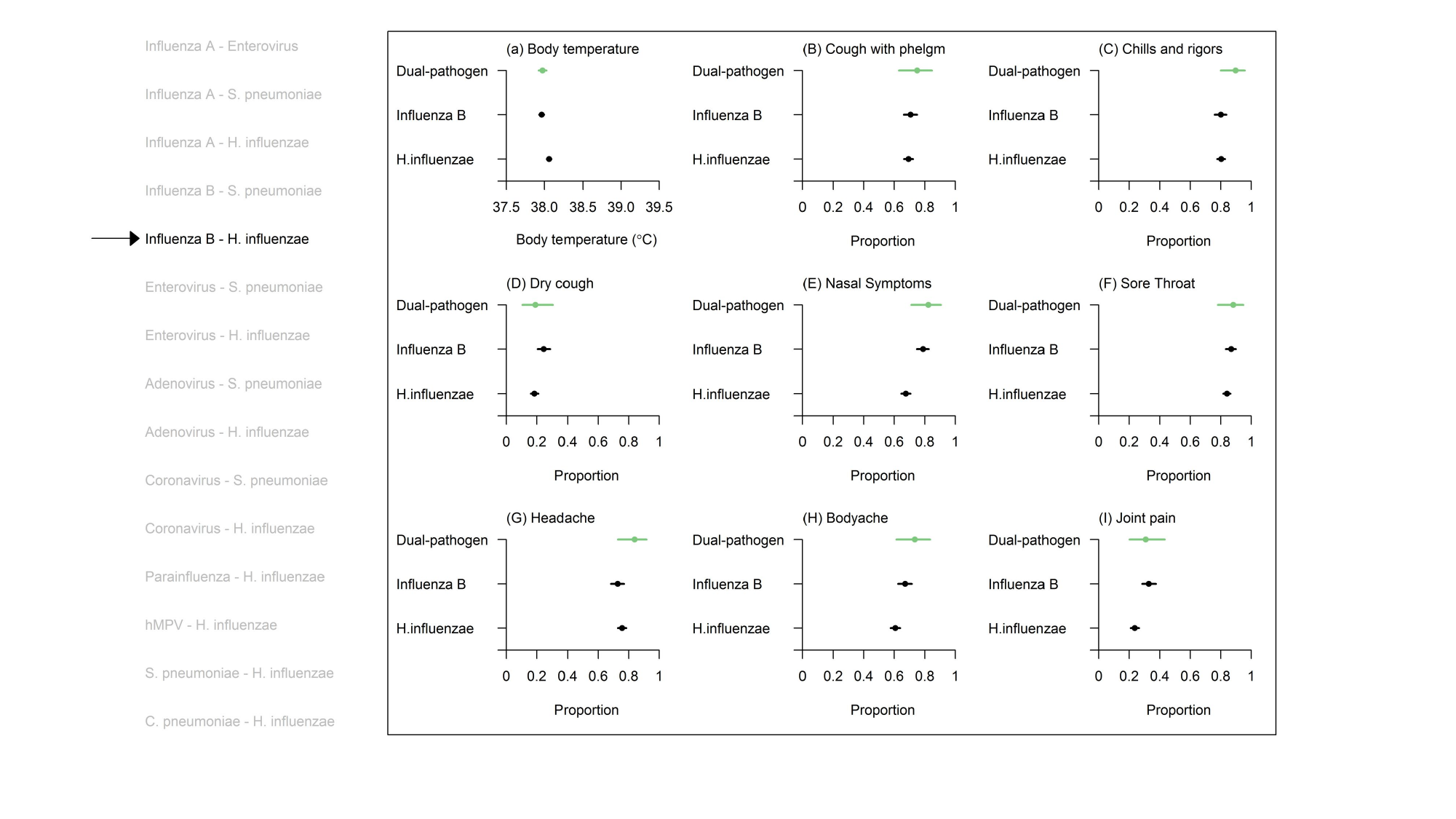

## Slide 6
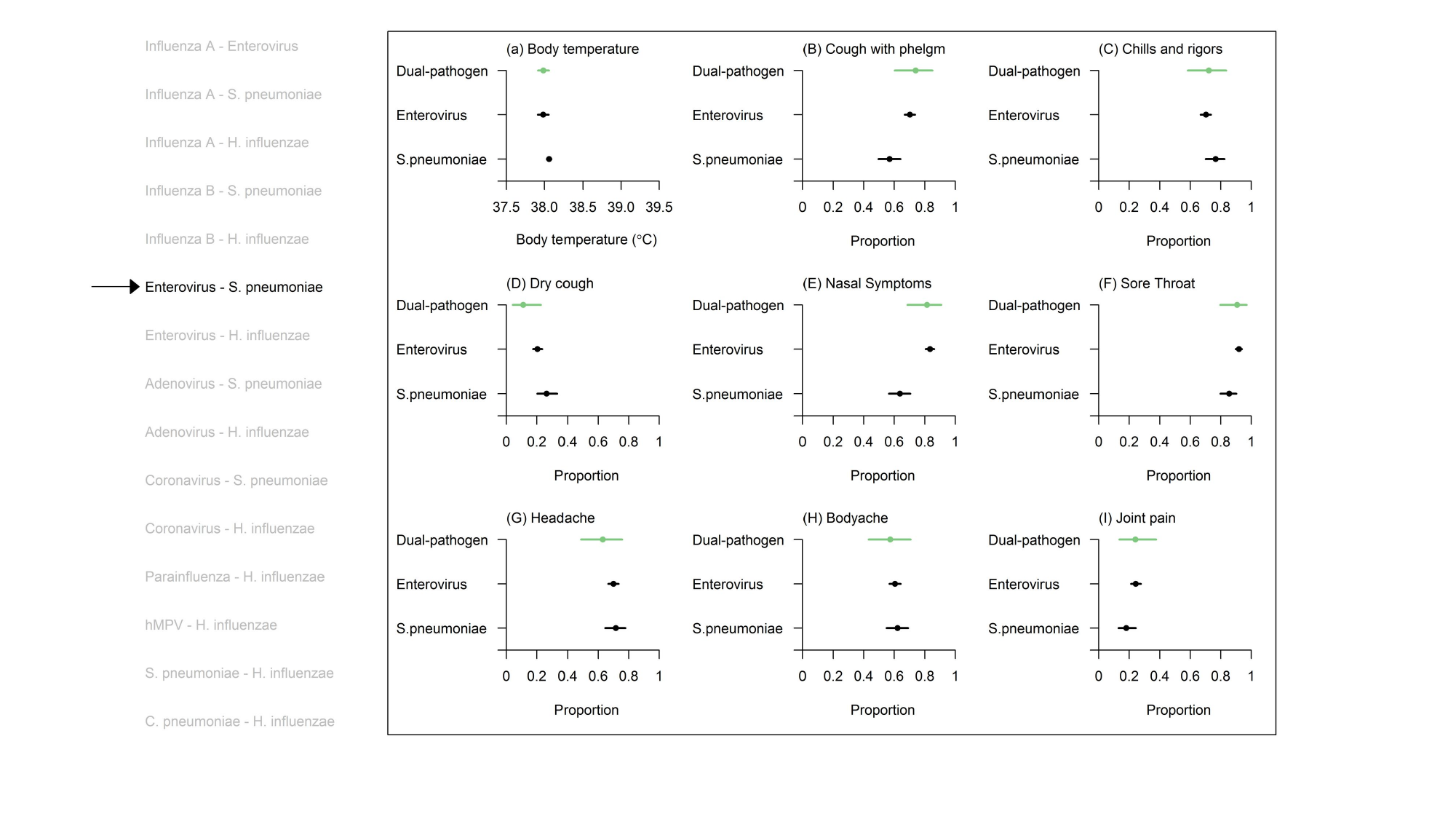

## Slide 7
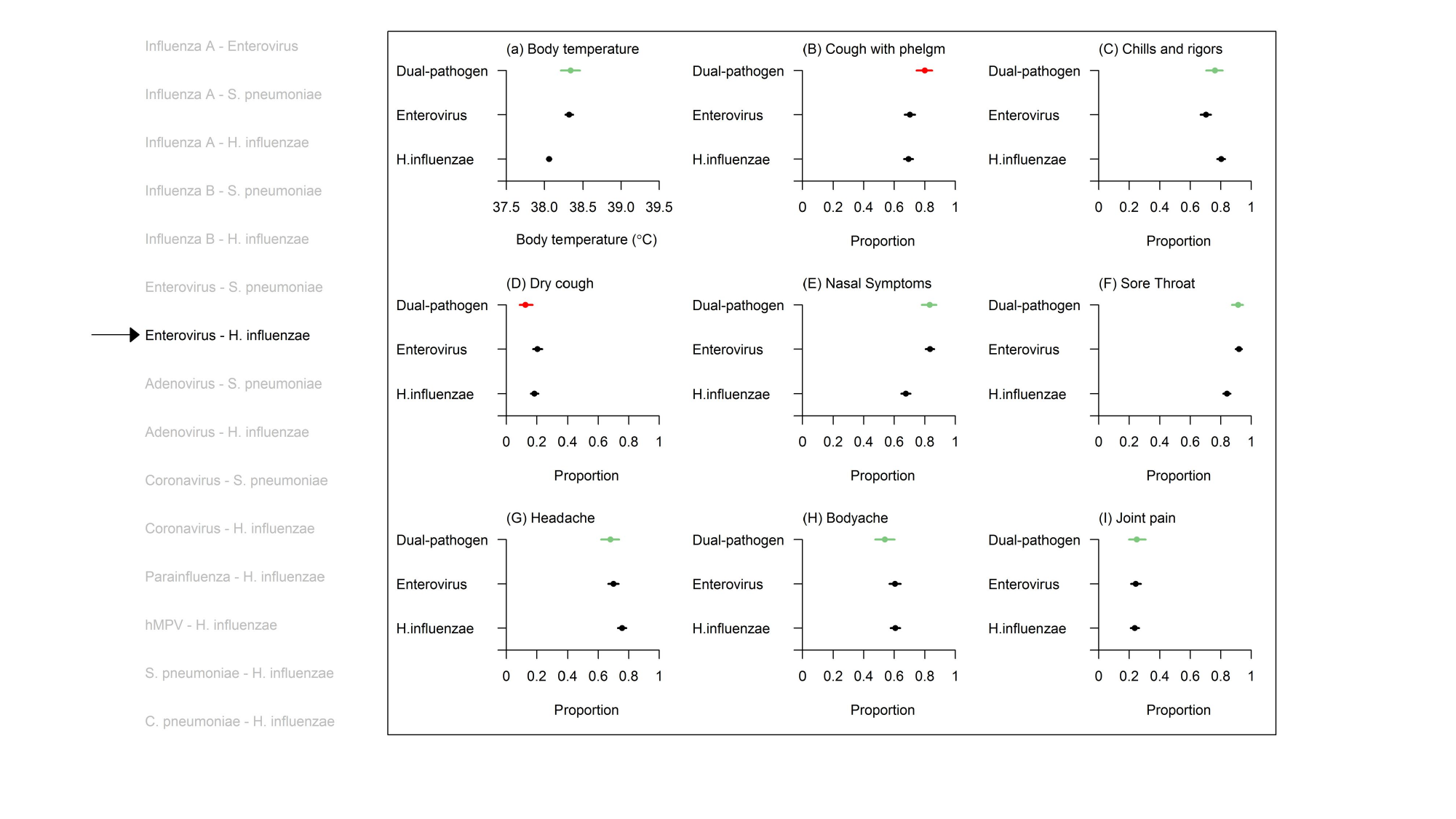

## Slide 8
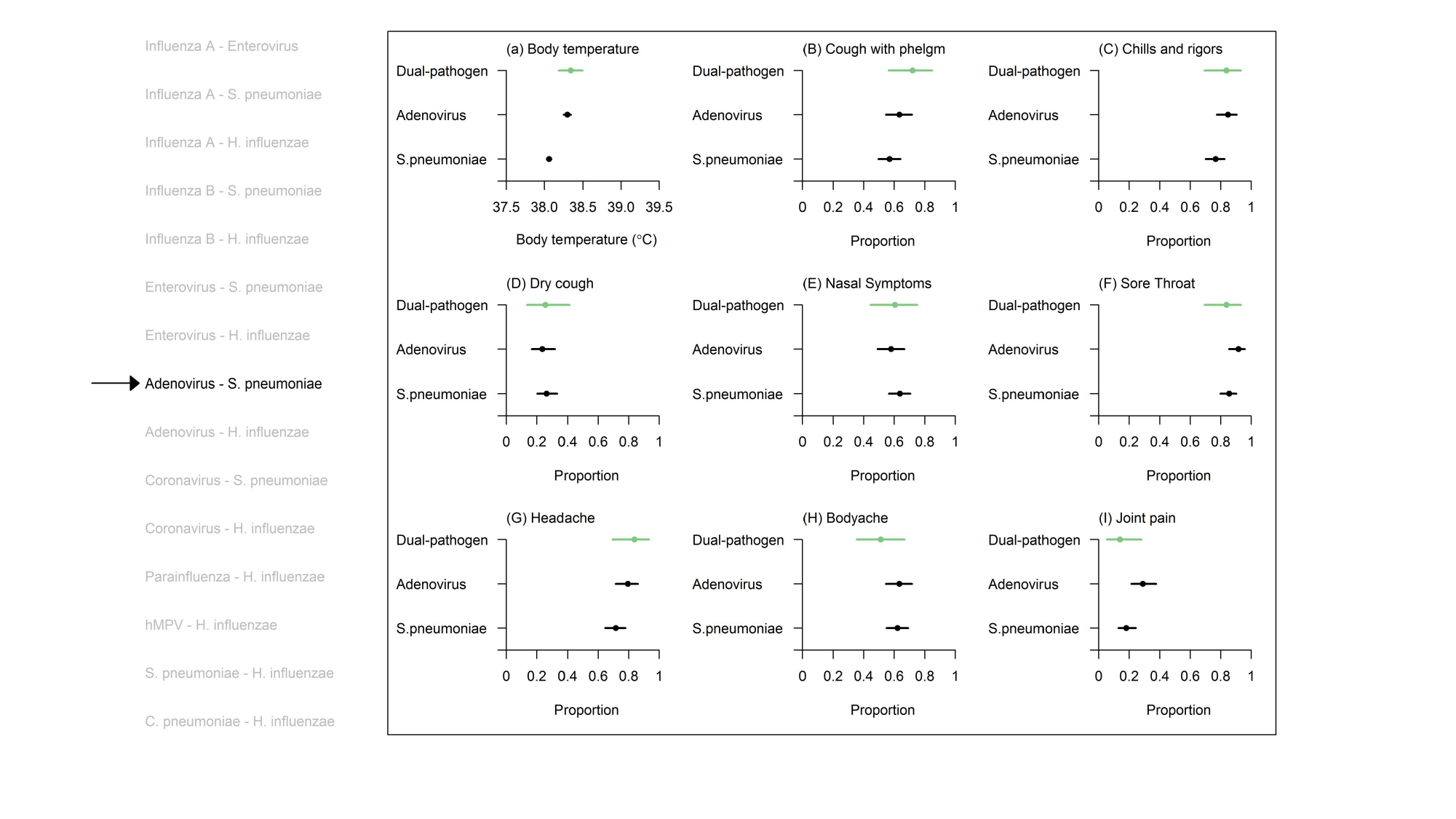

## Slide 9
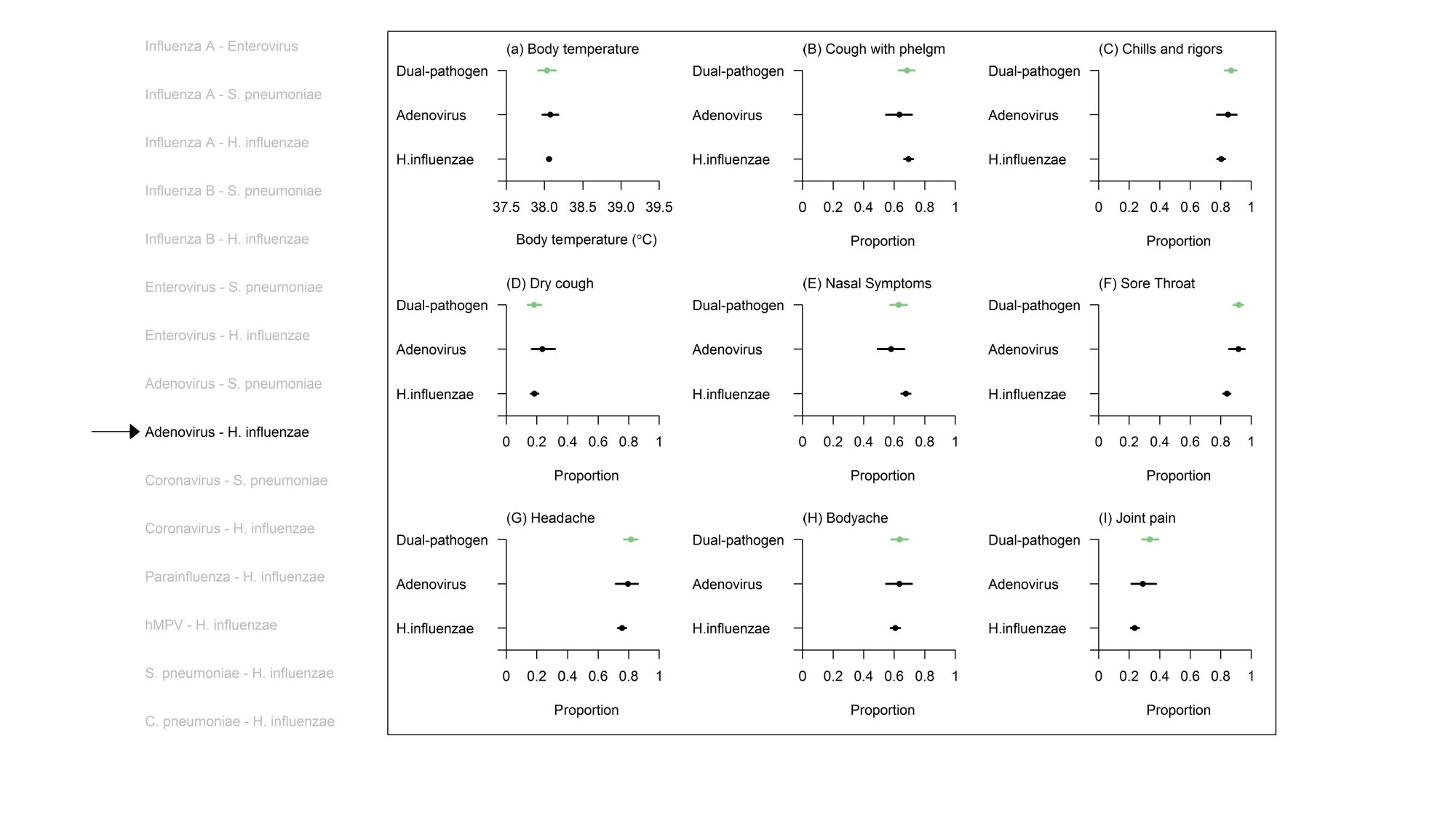

## Slide 10
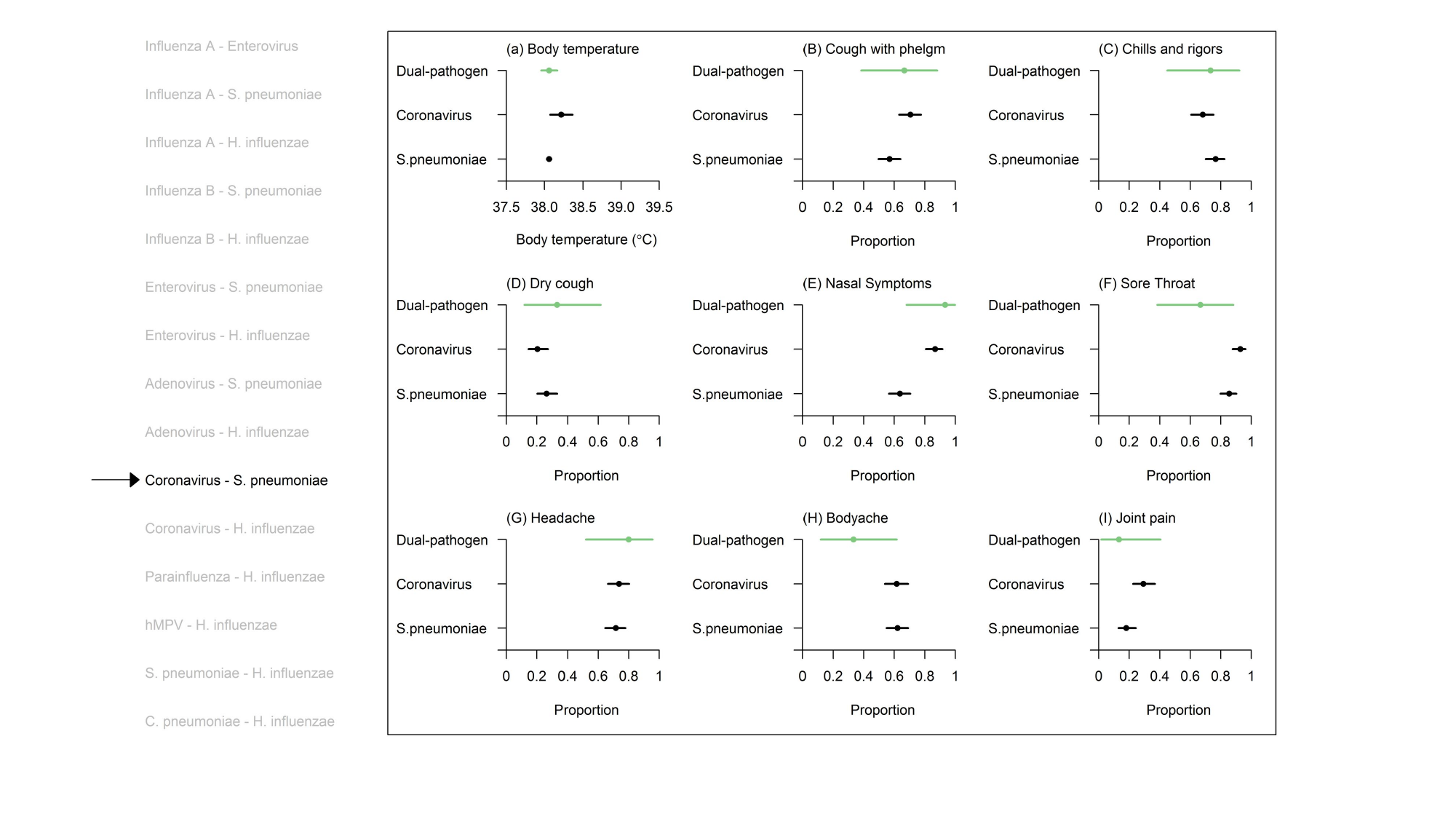

## Slide 11
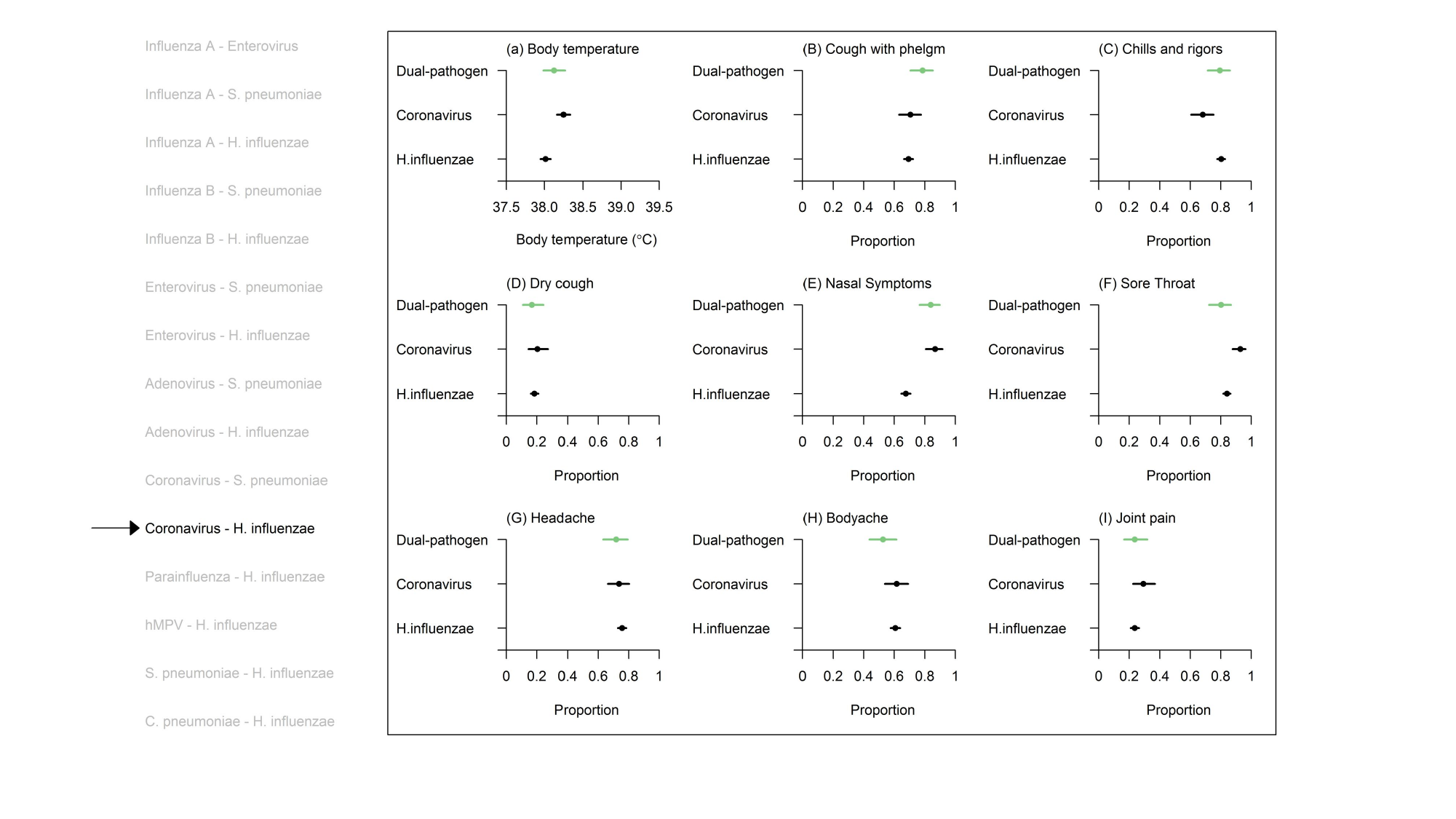

## Slide 12
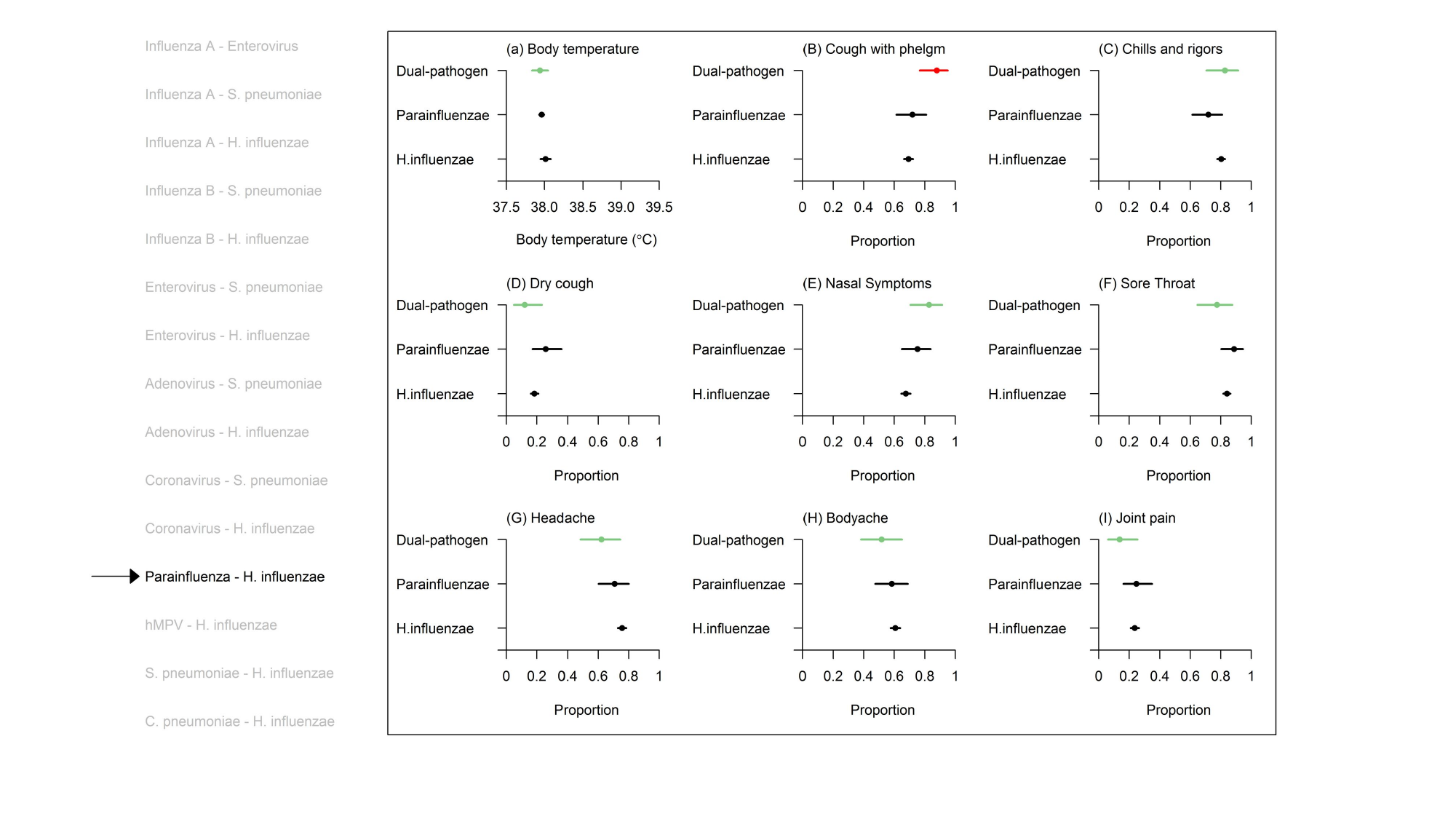

## Slide 13
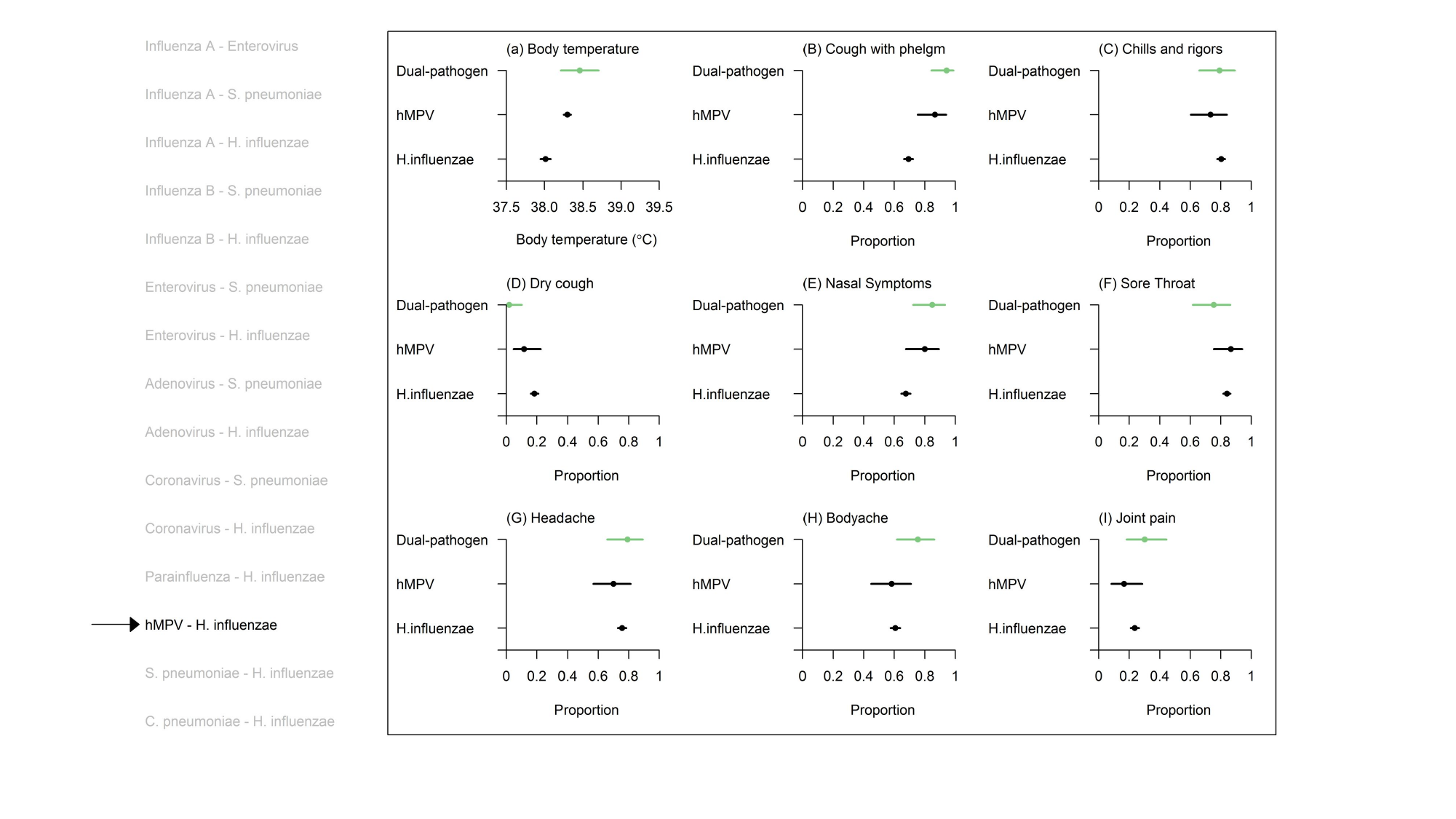

## Slide 14
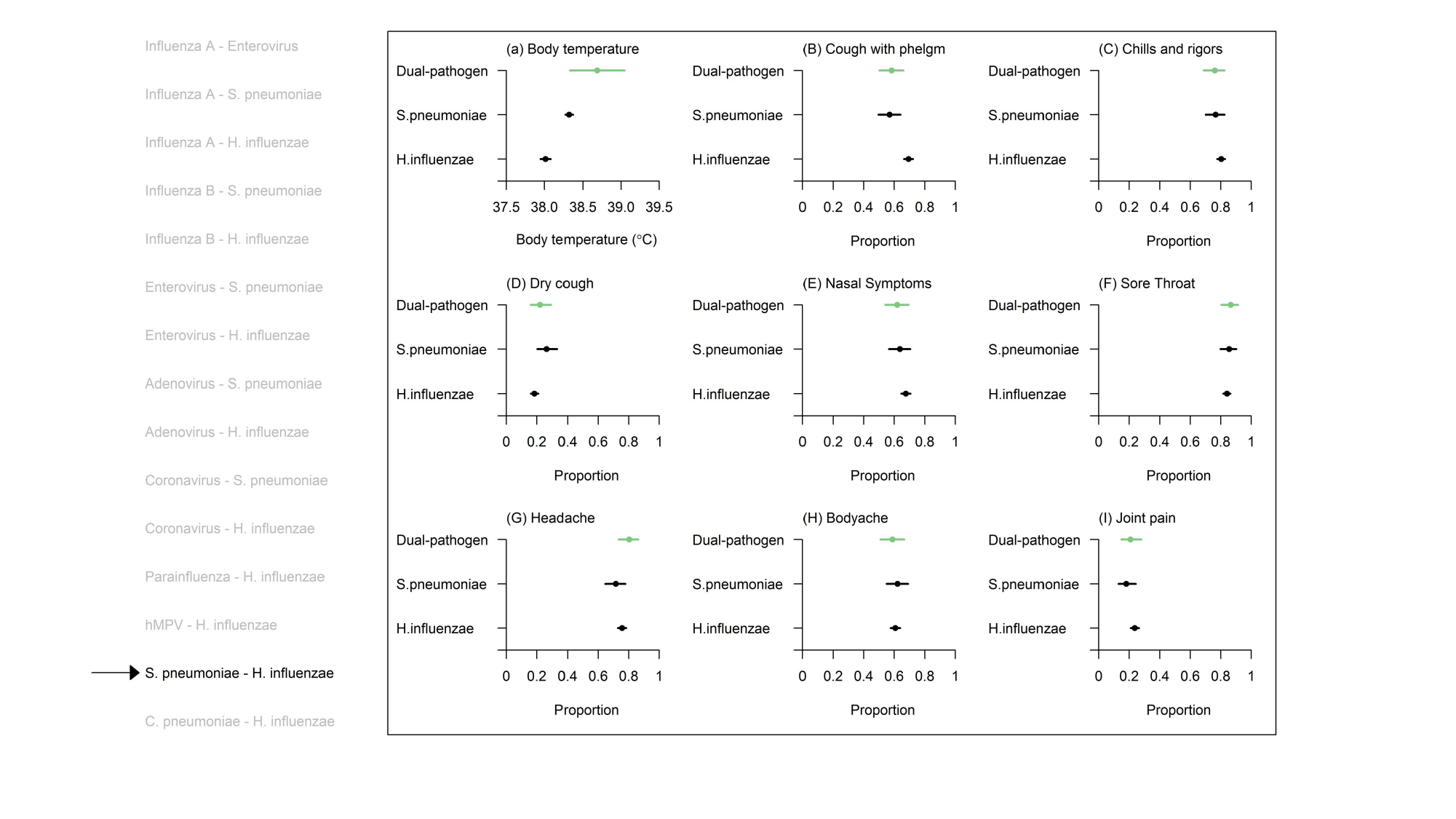

## Slide 15
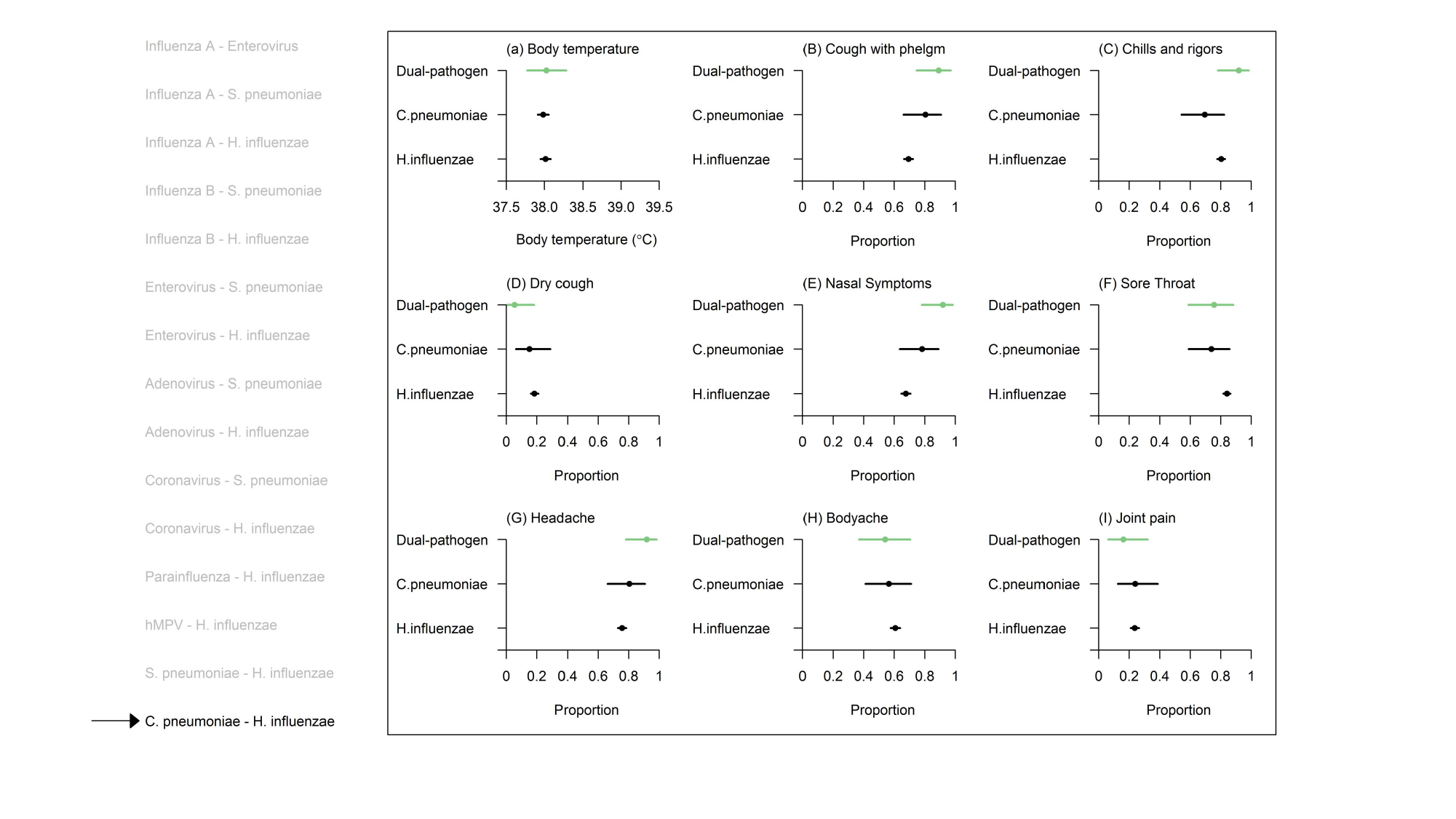

Supplement: Supplementary file 1 [file irv0009-0200-sd1.pptx]
