# Supplementary material for: Clinical differences between respiratory viral and bacterial mono- and dual pathogen detected among Singapore military servicemen with febrile respiratory illness
Source: Influenza Other Respir Viruses. 2015 Jun 9;9(4):200–8. doi: 10.1111/irv.12312 (PMC4474496; doi:10.1111/irv.12312)
Supplement: Supplementary file 2 [file irv0009-0200-sd2.docx]

**SUPPLEMENTARY INFORMATION**

|  | **Flu A** | **Flu B** | **RSV** | **Parainf-luenzae** | **hMPV** | **Entero-virus** | **Adeno-virus** | **Corona-virus** | **M. pneumo** | **C. pneumo** | **S. pneumo** | **N. mening** | **H. influenz** |
| --- | --- | --- | --- | --- | --- | --- | --- | --- | --- | --- | --- | --- | --- |
| **Flu A** |  | 5 | 0 | 4 | 4 | **20** | 1 | 11 | 0 | 2 | **17** | 7 | **63** |
| **Flu B** | 5 |  | 1 | 3 | 3 | **19** | 6 | 8 | 0 | 0 | **15** | 12 | **68** |
| **RSV** | 0 | 1 |  | 1 | 0 | 0 | 1 | 0 | 0 | 0 | 1 | 2 | 3 |
| **Parainfluenzae** | 4 | 3 | 1 |  | 0 | 6 | 1 | 5 | 0 | 1 | 6 | 4 | **58** |
| **hMPV** | 4 | 3 | 0 | 0 |  | 3 | 3 | 0 | 0 | 0 | 4 | 1 | **53** |
| **Entero-virus** | **20** | **19** | 0 | 6 | 3 |  | 5 | **15** | 0 | 0 | **68** | **84** | **160** |
| **Adeno-virus** | 1 | 6 | 1 | 1 | 3 | 5 |  | 3 | 1 | 9 | **43** | 13 | **319** |
| **Corona-virus** | 11 | 8 | 0 | 5 | 0 | **15** | 3 |  | 0 | 3 | **15** | 5 | **131** |
| **M. pneumo** | 0 | 0 | 0 | 0 | 0 | 0 | 1 | 0 |  | 2 | 0 | 0 | 2 |
| **C. pneumo** | 2 | 0 | 0 | 1 | 0 | 0 | 9 | 3 | 2 |  | 3 | 6 | **37** |
| **S. pneumo** | **17** | **15** | 1 | 6 | 4 | **68** | **43** | **15** | 0 | 3 |  | 9 | **163** |
| **N. mening** | 7 | 12 | 2 | 4 | 1 | **84** | 13 | 5 | 0 | 6 | 9 |  | 14 |
| **H. influenz** | **63** | **68** | 3 | **58** | **53** | **160** | **319** | **131** | 2 | **37** | **163** | 14 |  |

**Table S1. Checkerboard of dual-pathogens detected among cases.**  The 18 dual-pathogen pairs for further analysis of symptoms (i.e. 15 observations or more) are in bold.

| **Symptom** | **Mean Proportions [95%CI]**  **(unless otherwise stated)** | | | ***Adjusted P-Value*** | | |
| --- | --- | --- | --- | --- | --- | --- |
|  | **Viral Mono-Pathogen**  **(n = 2235)** | **Bacterial Mono-Pathogen**  **(n = 1305)** | **Dual-Pathogens**  **(n = 1564)** | ***Viral vs Bacterial Mono-Pathogen*** | ***Viral Mono- Pathogen vs Dual-Pathogen*** | ***Bacterial Mono-Pathogen vs Dual-Pathogen*** |
| Body Temperature (^ᵒ^C) | 38.2  [38.1, 38.2] | 38.1  [38.0, 38.1] | 38.1  [38.1, 38.1] | *<0.001* | *0.002* | *0.04* |
| Chills and Rigors | 0.74  [0.72,0.76] | 0.79  [0.77,0.81] | 0.82  [0.80,0.84] | *<0.001* | *<0.001* | *0.24* |
| Cough with Sputum | 0.68  [0.66,0.70] | 0.68  [0.65,0.70] | 0.74  [0.72,0.76] | *1.00* | *<0.001* | *<0.001* |
| Dry Cough | 0.25  [0.23,0.27] | 0.19  [0.17,0.22] | 0.17  [0.15,0.19] | *<0.001* | *<0.001* | *0.39* |
| Nasal Symptoms | 0.79  [0.77,0.81] | 0.67  [0.64,0.70] | 0.75  [0.73,0.77] | *<0.001* | *0.01* | *<0001* |
| Sore Throat | 0.87  [0.85, 0.88] | 0.84  [0.82,0.86] | 0.87  [0.85,0.88] | *0.04* | *1.00* | *0.11* |
| Headache | 0.73  [0.71,0.75] | 0.75  [0.72,0.77] | 0.76  [0.74,0.79] | *0.62* | *0.03* | *0.88* |
| Body Ache | 0.64  [0.62,0.66] | 0.61  [0.58,0.64] | 0.60  [0.58,0.63] | *0.35* | *0.09* | *1.00* |
| Joint Pain | 0.30  [0.28,0.32] | 0.23  [0.21,0.25] | 0.26  [0.24,0.29] | *<0.001* | *0.06* | *0.11* |

**Table S2. Mean proportions and Comparisons between Viral and Bacterial Mono-pathogens and Dual pathogens.** Nasal symptoms include sneezing, blocked nose and running nose. Means were compared using ANOVA test, while proportions were compared using Pearson’s Chi-square test. P-values were adjusted using Bonferroni correction.
